# Automated Interpretation of Lung Sounds by Deep Learning in Children With Asthma: Scoping Review and Strengths, Weaknesses, Opportunities, and Threats Analysis

**DOI:** 10.2196/53662

**Published:** 2024-08-23

**Authors:** Isabelle Ruchonnet-Métrailler, Johan N Siebert, Mary-Anne Hartley, Laurence Lacroix

**Affiliations:** 1 Pediatric Pulmonology Unit Department of Pediatrics Geneva Children’s Hospital, University Hospitals of Geneva Geneva Switzerland; 2 Faculty of Medicine University of Geneva Geneva Switzerland; 3 Division of Pediatric Emergency Medicine, Department of Pediatrics Geneva Children’s Hospital Geneva University Hospitals Geneva Switzerland; 4 Intelligent Global Health Research Group Machine Learning and Optimization Laboratory Swiss Federal Institute of Technology Lausanne Switzerland; 5 Laboratory of Intelligent Global Health Technologies, Bioinformatics and Data Science Yale School of Medicine New Haven, CT United States

**Keywords:** asthma, wheezing disorders, artificial intelligence, deep learning, machine learning, respiratory sounds, auscultation, stethoscope, pediatric, mobile phone

## Abstract

**Background:**

The interpretation of lung sounds plays a crucial role in the appropriate diagnosis and management of pediatric asthma. Applying artificial intelligence (AI) to this task has the potential to better standardize assessment and may even improve its predictive potential.

**Objective:**

This study aims to objectively review the literature on AI-assisted lung auscultation for pediatric asthma and provide a balanced assessment of its strengths, weaknesses, opportunities, and threats.

**Methods:**

A scoping review on AI-assisted lung sound analysis in children with asthma was conducted across 4 major scientific databases (PubMed, MEDLINE Ovid, Embase, and Web of Science), supplemented by a gray literature search on Google Scholar, to identify relevant studies published from January 1, 2000, until May 23, 2023. The search strategy incorporated a combination of keywords related to AI, pulmonary auscultation, children, and asthma. The quality of eligible studies was assessed using the ChAMAI (Checklist for the Assessment of Medical Artificial Intelligence).

**Results:**

The search identified 7 relevant studies out of 82 (9%) to be included through an academic literature search, while 11 of 250 (4.4%) studies from the gray literature search were considered but not included in the subsequent review and quality assessment. All had poor to medium ChAMAI scores, mostly due to the absence of external validation. Identified strengths were improved predictive accuracy of AI to allow for prompt and early diagnosis, personalized management strategies, and remote monitoring capabilities. Weaknesses were the heterogeneity between studies and the lack of standardization in data collection and interpretation. Opportunities were the potential of coordinated surveillance, growing data sets, and new ways of collaboratively learning from distributed data. Threats were both generic for the field of medical AI (loss of interpretability) but also specific to the use case, as clinicians might lose the skill of auscultation.

**Conclusions:**

To achieve the opportunities of automated lung auscultation, there is a need to address weaknesses and threats with large-scale coordinated data collection in globally representative populations and leveraging new approaches to collaborative learning.

## Introduction

### Background

Asthma is one of the most common chronic pediatric conditions in the world, affecting almost 10% of children and causing significant morbidity and mortality [1], as well as putting a considerable burden on health care systems [1]. It is the third leading cause of admission among children aged <15 years [2]. In total, 7% of children with asthma (aged <18 years) require hospital admission each year due to exacerbation episodes, 47% of children have sleep disturbances or show limitation in daily activities [3], and 25% of children report increased school absences [4]. Mismanagement of the condition can cause long-lasting pulmonary sequelae with decreased lung function persisting into adulthood [5].

Asthma management is often delegated to patients and caregivers, who estimate treatment requirements according to their subjective understanding of symptom severity. The presence of various inevitable confounding factors may lead to poorly adapted and inequitable treatment. Although a written, symptom-based action plan for patients and caregivers is recommended to improve asthma control and avoid hospitalization, it is not sufficient to significantly reduce the risk for subsequent pediatric emergency department visits [6]. Careful monitoring and management of acute exacerbation episodes and long-term treatment are therefore essential to decrease morbidity and mortality and to improve the quality of life of children with asthma and their families.

Since the invention of the stethoscope by René Laennec in 1816, pulmonary auscultation has become a fundamental part of routine physical examination. However, the interpretation of sounds is ultimately limited by the human capacity for auditory perception and cognitive pattern detection. Recent efforts to digitalize auscultation audio allow the recording of sounds that can then be exploited by various software to assist interpretation [7]. A particular advantage is that recordings capture a higher resolution and wider range of frequencies, which are not usually perceptible to the average (and especially, the aging) human ear (ie, outside 20-20,000 Hz). The digital information obtained from the sounds recorded by electronic auscultation can, in turn, inform computer software, aiming to overcome standard lung auscultation limitations by removing subjectivity, standardizing interpretation through objective, nuanced acoustic pattern recognition, and automating the identification of abnormal auscultations indicating respiratory diseases. Recent advances in signal processing and computational efficiency offer the promise of accessible high-performance analysis for many physiological measurements [7]. In particular, deep learning (commonly referred to as artificial intelligence [AI]) opens up new perspectives to improve diagnostic accuracy and enable the automated monitoring of respiratory diseases by nonphysicians [8,9]. Studies showing this potential have mostly focused on adults [10]. The few studies that focused on pediatric auscultation [11-13] highlighted the high potential impact of standardizing the identification and assessment of obstructive pulmonary sounds, which are known reliable estimators of the severity and treatment response in asthma and wheezing disorders. Promising results have emerged in the automated detection of wheezing disorders and pneumonia [12,13]. Even early iterations in 2008 showed automated recognition of wheezing to be superior to traditional lung auscultation performed by untrained or trained medical staff in outpatient or intensive care units [14]. Beyond improved diagnosis and management, there is the potential to decentralize lung auscultation to home-based monitoring and remote care for earlier detection of asthma exacerbations, potentially reducing morbidity and health care costs [15].

### Objective

The purpose of this scoping review is to assess the state of the art of the research conducted in AI applied to digital lung sound analysis in children with asthma and provide a balanced assessment of its strengths, weaknesses, opportunities, and threats (SWOT). To our knowledge, this is the first such review and critical assessment on the topic.

## Methods

### Review Question

On the basis of the Population targeted, Concept under investigation, Context parameters framework [16], the review question driving the search strategy was “How has AI-driven automated lung sound analysis been used to detect or classify asthma and wheezing disorders in children over the past 23 years (2000-2023), considering its strengths, weaknesses, opportunities, and threats?” (Multimedia Appendix 1).

### Information Sources and Search Strategy

The review complies with the PRISMA-ScR (Preferred Reporting Items for Systematic Reviews and Meta-Analyses extension for Scoping Reviews) [17]. An electronic literature search was performed independently by 2 of the authors with expertise in pediatric emergency medicine and pulmonology (JNS and IR-M). The search was initially focused on academic literature in PubMed, MEDLINE Ovid, Embase, and Web of Science databases, with the date range restricted between January 1, 2000, and May 23. In addition, to broaden the scope, gray literature was sought using Google Scholar with the search limited to the first 10 pages of results (ie, first 250 occurrences) [18,19]. Search terms were chosen to filter out articles on AI-based lung sound analysis for the detection of asthma in humans. The following medical subject headings were explored and connected by Boolean operators with adjustments in syntax adapted to individual databases: artificial intelligence; machine learning; deep learning; neural networks, computer; respiratory sounds; asthma; wheezing disorder. The articles retrieved were further refined for pediatric studies using the following keywords: “adolescent” OR “children” OR “child, preschool” [20] OR “infant” (Table 1).

**Table 1:** Search strategy.

Database and filter			Articles, n
**PubMed**			
	Artificial intelligence OR machine learning OR deep learning OR neural networks, computer	262,346	
	AND respiratory sounds	283	
	AND (asthma OR wheezing disorder)	147	
	AND (adolescent OR children OR child, preschool OR infant)	32	
**MEDLINE Ovid**			
	(Artificial intelligence or machine learning or deep learning or neural networks, computer).mp	94,359	
	AND respiratory sounds.mp	132	
	AND (asthma OR wheezing disorder).mp	25	
	AND (adolescent OR children OR child, preschool OR infant).mp	16	
**Embase**			
	(“artificial intelligence”/exp OR “artificial intelligence” OR (artificial AND (“intelligence”/exp OR intelligence)) OR “machine learning”/exp OR “machine learning” OR (“machine”/exp OR machine) AND (“learning”/exp OR learning)) OR “deepl learning” OR (deepl AND (“learning”/exp OR learning)) OR “neural networks, computer”/exp OR “'neural networks, computer’” OR (neural AND networks, AND (“computer”/exp OR computer))) AND [humans]/lim AND [english]/lim AND [2000-2023]/py	154,919	
	AND respiratory sounds	222	
	AND (asthma OR wheezing disorder)	43	
	AND (adolescent OR children OR child, preschool OR infant)	13	
**Web of Science**			
	TS=(Artificial intelligence OR machine learning OR deep learning OR neural networks, computer) and DOP=(200-01-01/2023-05-23) AND LA=(English)	431,019	
	AND TS=(respiratory sounds)	183	
	AND TS=(asthma OR wheezing disorder)	35	
	AND TS=(adolescent OR children OR child, preschool OR infant)	11	
**Google Scholar (gray literature)**			
	Artificial intelligence OR machine learning OR deep learning OR neural networks	129,000	
	AND respiratory sounds	18,800	
	AND (asthma OR wheezing disorder)	15,300	
	AND (adolescent OR children OR infant)-cough	8720	
	Initial occurrences^a^	250	

^a^Consistent with recommendations to prioritize the first 200 to 300 results in article titles when searching for gray literature [18], the initial 250 occurrences were retained.

### Eligibility Criteria

Identified articles were submitted to inclusion criteria for eligibility: articles should have described automated lung sound analysis in children from birth to 18 years of age with asthma (Textbox 1). Articles should have been written in English. Articles were excluded if the study was conducted on animals. Studies concerning cough rather than lung sounds were excluded. Comments on articles and unpublished work were not considered suitable and were excluded from the review.

**Population, Intervention, Comparator, Outcome, and Study design (PICOS framework) eligibility criteria for the scoping review.**

**Inclusion criteria**

- Population
  Infants, children, and adolescents aged <18 years
  Asthma or wheezing disorder
- Intervention
  Digital auscultation coupled with automated lung sound analysis using artificial intelligence to detect or classify asthma
  Audio files specifically collected for the purpose of the study or from a publicly available database
- Comparator
  Asthma lung sounds
- Outcome
  Predictive measures of performance
- Study design
  Research articles
- Period
  January 1, 2000, to May 23, 2023
- Language
  English

**Exclusion criteria**

- Population
  Adult population (aged ≥18 years)
  Other diseases causing abnormal respiratory sounds
- Intervention
  No artificial intelligence used
  Audio files retrieved solely from lung sound databases, which exhibit multiple acquisition flaws inducing systematic biases between predicted labels (eg, disparate diagnoses sourced from various locations, systematically diverse age groups, or using different stethoscopes)
- Comparator
  Normal or pathological lung sounds other than those associated with asthma
  Cough or voice sounds
- Outcome
  No performance metric report
- Study design
  Abstracts
  Book chapters
  Communications
  Commentaries to articles
  Conference papers
  Letters to the editor
  Study protocols
  Thesis
  Unpublished work
- Period
  Outside date range
- Language
  Other than English

### Selection of Sources of Evidence

#### Academic Literature Search

The articles retrieved from the 4 academic repositories were merged, and duplicates were removed. Any relevant article with a title or abstract including the search terms was independently screened by the same 2 reviewers, who completed the database search process. Disagreements were resolved by consensus with a third reviewer (LL). The reference lists of eligible articles were reviewed to find additional relevant articles and the related papers obtained. This procedure was repeated until the reference lists covered the complete set of articles already retrieved.

#### Gray Literature Search

Gray literature was screened by one of the reviewers (JNS). Given the inherent content variability and nonpeer-reviewed status of the sources included, gray literature search was excluded from the subsequent quality assessment. This decision was made to maintain consistency in the assessment process and ensure alignment with established quality evaluation criteria applied to peer-reviewed literature. In addition, the reference lists of the identified articles were not reviewed.

### Data Charting

Data from the selected articles obtained through academic literature search were extracted by one of the authors (JNS) in the following structured format: publication reference; number of participants and conditions when available, age, number of recordings; study design; number of classes; methodology; recording device; AI model architecture used; findings and performance (metrics) of the algorithm; advantages and limitations of the study; and sharing algorithm code or data as open source. As for the articles from the gray literature, they are provided in the following format: publication reference; article type; and a brief summary of the article.

### Critical Appraisal and Data Synthesis

Quality assessment of selected studies from the academic literature search was performed by JNS using the Checklist for the Assessment of Medical Artificial Intelligence (ChAMAI) [21] and then reviewed by LL. An agreement was reached through discussion. The ChAMAI is a 30-item practical assessment-oriented checklist that is recommended to be reported for medical AI studies to encourage reproducible results and evaluation. It aims to distinguish high-quality medical AI studies from the simple application of AI techniques to medical data. The checklist is organized into six dimensions according to the Cross Industry Standard Process for Data Mining methodology [22]: (1) problem understanding, (2) data understanding, (3) data preparation, (4) modeling, (5) validation, and (6) deployment. Each item can be rated as OK (adequately addressed), minor revision (mR; sufficient but requiring mR to improve), or major revision (MR; insufficiently addressed and necessarily requiring MR or justifying rejection of the manuscript). A total of 20 (67%) of the 30 high-priority items can be assigned a value of 2, 1, and 0 depending on their respective rating (OK, mR, or MR). For the remaining 10 (30%) low-priority items, the values are halved (ie, 1, 0.5, and 0). The maximum score is 50 points. Study quality was classified as low (0-19.5), medium (20-34.5), or high (35-50) [23].

### SWOT Analysis

A SWOT analysis categorizes factors describing a research subject into 4 distinct groups: strengths, weaknesses, opportunities, and threats. Strengths and weaknesses are internal factors controlled or influenced by the entity itself, such as its resources, capabilities, and organizational structure, while opportunities and threats are external factors beyond the entity’s direct control that can significantly impact its activity [24]. Although primarily used in organizational studies [25], we used SWOT as a structured analysis framework to conduct a balanced assessment of the SWOT associated with AI-assisted lung auscultation for pediatric asthma, drawing from the academic literature retrieved in this scoping review.

## Results

### Selection of Sources of Evidence

**Figure 1:**
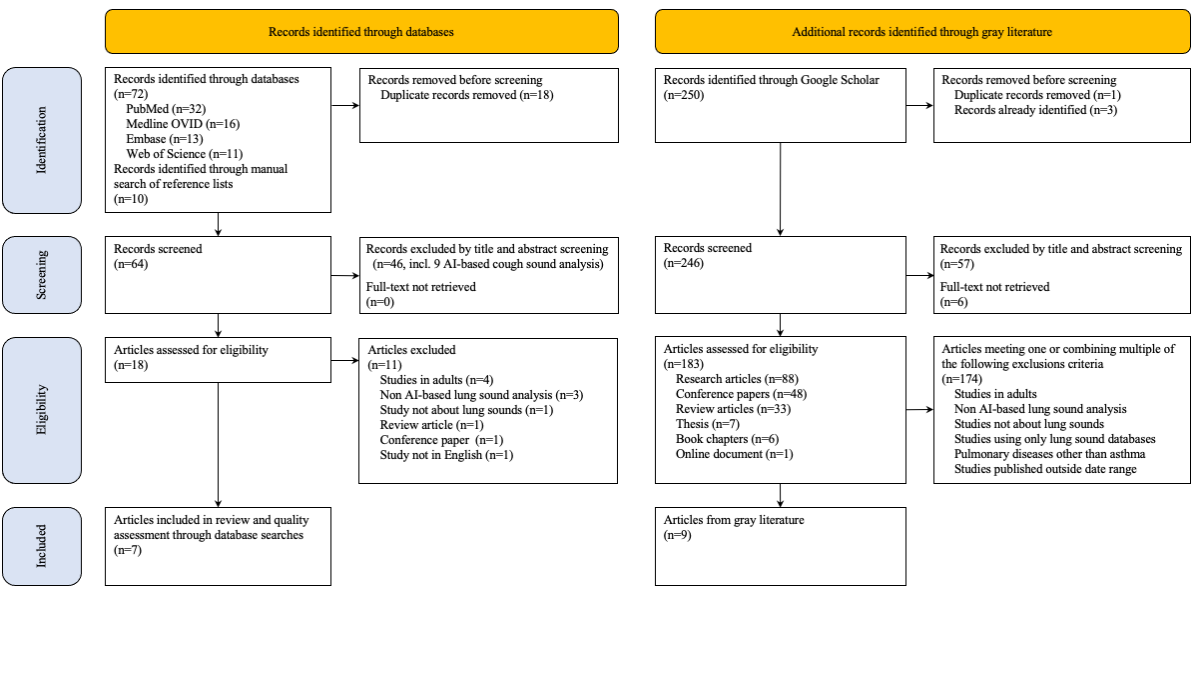
PRISMA-ScR (Preferred Reporting Items for Systematic Reviews and Meta-Analyses guidelines extension for Scoping Reviews) flowchart depicting included studies from academic databases and gray literature.

The search strategy selected 82 articles from academic database searches, of which 64 (78%) were only screened, and 18 (22%) were assessed for full-text eligibility. In addition, 250 records were retrieved from gray literature search, with 183 (73%) assessed for eligibility, resulting in a total of 201 unique records (Figure 1). Of the 46 articles not included at the screening stage, 9 (20%) pertained to AI-based asthma or wheezing recognition algorithms using cough sounds [26-34]. Of the 18 studies, 11 (61%) identified through the academic literature search were excluded due to ineligible study design (n=4, 36% in adults; n=4, 36% not relying on AI or lung sounds; and n=3, 27% other study types). Among them were 3 quasi-experimental studies [35-37] focused on computerized wheeze detection through respiratory spectrum analysis in children with asthma, not involving the support of AI algorithms. Along with records from the gray literature search, a total of 9% (7/82) and 3.6% (9/250) of studies were respectively included in the scoping review.

### Characteristics of Individual Sources of Evidence

The characteristics of the 7 studies included from the academic literature search are presented in Table 2. They were conducted between 2015, and 2022, across 8 different countries (Croatia, Poland, Australia, China, Singapore, the Republic of Korea, and Russia). With the exception of the study by Mazić et al [38], which was performed in a general hospital, all AI models were implemented in children’s hospitals, either in respiratory clinics or inpatient wards. Study participants varied in number, ranging from 16 to 112. The participant count was not specified in 1 study by Gelman et al [39], involving individuals aged 28 days to 18 years. The design of the studies was also variable, with 4 quasi-experimental studies and 3 prospective cohort studies. Lung sounds were collected using a variety of custom-built [38,40] and commercial (StethoMe, Littmann 3200, CliniCloud, Yunting model II, and Jabes) digital stethoscopes (including a mix of various stethoscopes in 1 of the studies) [11,12,41,42], or with built-in or external cell phone microphones [39]. Although most of the studies used patients’ lung sounds under realistic conditions in their AI models, the study by Cheng et al [40] also incorporated breath sounds from a commercially available database.

**Table 2:** Characteristics of individual sources of evidence from academic literature search.

Study	Participants, N^a^ (age; recordings, n)	Study design	Classes, n	Methodology	Recording device	AI^b^ model architecture	Findings, metrics	Advantages and limitations	Open- source
Mazić et al [38]	16 with obstructive bronchitis or asthma (1-6 y, 45)	Quasi-experimental	2: wheezes and nonwheezes	AI-based binary classifier development using recordings from hospital visits in Croatia under realistic conditions; no lung sound database	Accelerometer	2 cascaded SVM^c^ classifiers	A 2-layer parallel-stacked SVM classifier enhances the standard SVM classifier with high reliability of wheezing recognition: accuracy 97.7%	Advantages: cascade classifier increases accuracy Limitations: no gold standard. Validation of system not reported. No wheezing in the testing set. A small number of patients. No healthy subjects. No tabular data	No code and no data
Grzywalski et al [11]	50 (1-18 y; 522 total: 124 wheezes, 113 rhonchi, 66 coarse crackles, and 112 fine crackles)	Prospective cohort study	4: wheezes, rhonchi, and fine and coarse crackles	Recordings of Polish inpatients under realistic conditions. No lung sound database. Five pediatricians classified recordings as gold standard. Comparison: 5 pediatricians versus AI in detecting 4 pathological sound types	Digital stethoscope (Littmann3200)	CNNs^d^ and StethoMe AI^e^	The AI model slightly outperforms pediatrician auscultations: accuracy 57.7% versus 66.1%, sensibility 78.2% versus 58.1%, specificity 82.2% versus 90.7%, and *F*_1_-score 66.4% versus 61.8%	Advantages: establishment of a gold standard. Varied pediatric age range Limitations: human labeling subjectivity. Training and validation set undisclosed. No healthy participants. No tabular data	No code and no data
Kevat et al [12]	25 (median 6.7 y; 192 total: 40 wheezes, 39 wheezes+crackles, 46 cystic fibrosis unspecified sounds, and 67 normal breath sounds)	Quasi-experimental	3: wheezes, crackles, and normal breath sounds	Recordings of Australian inpatients in Australia under realistic conditions. No lung sound database. Classified by 1 pediatric pulmonologist	Digital stethoscopes (Clinicloud and Littman 3200)	CNNs and StethoMe AI	The AI model consistently identified pediatric breath sounds across various devices, while variations based on device type may be present: Clinicloud: sensibility 90% and specificity 97%; Littman: sensibility 80% and specificity 97%	Advantages: AI blinded to tagged recordings Limitations: no true gold standard. Human labeling subjectivity. Age range of participants not reported. Training and validation sets undisclosed. Reliance on 2 stethoscopes. No tabular data	No code, No data
Zhang et al [41]	112, including 5 with asthma, 15 with bronchitis, 15 with bronchiolitis, 1 with bronchiolitis obliterans, 75 with pneumonia, 1 with foreign body aspiration (28 d-18 y; 627 total: 204 wheezes, 159 crackles, and 264 normal breath sounds)	Quasi-experimental	3: wheezes, crackles, and normal breath sounds	Recordings of Chinese inpatients under realistic conditions. No lung sound database. Classified by 2 pediatric pulmonologists as gold standard. Comparison: 6 general pediatricians with over 5 years of experience versus AI in detecting 3 breath sound types	Digital stethoscope (Yunting model II)	SVM	The AI model outperforms general pediatrician auscultations: accuracy 76% versus 64.9%, sensibility 86.4% versus 82.2%, specificity 83% versus 72.1%, and *F*_1_-score 80.9% versus 72.5%	Advantages: establishment of a gold standard. Training and validation set disclosed. Varied pediatric age range Limitations: human labeling subjectivity. Few breath sounds of children aged >60 months. Cannot detect crackles and wheezes simultaneously, impacting diagnosis. No tabular data	No code and no data^f^
Cheng et al [40]	73, including 19 with viral wheeze or asthma exacerbation, 17 with well-controlled asthma, 2 with exacerbations of chronic lung disease, 8 with pneumonia, 3 with other conditions, 24 healthy children (5 mo-16 y; 73 total: 18 wheezes, 10 crackles, and 45 normal breath sounds)	Quasi-experimental	3: wheezes, crackles, and normal breath sounds	Recordings of outpatients and inpatients in Singapore under realistic conditions. Use of the RALE lung sound database [43]. Classified by 2 pediatric pulmonologists	Custom-built digital stethoscope with a smartphone	SVM	Suggest a ternary classification model for discriminating among wheezes, crackles, and normal breath sounds: sensibility 91% and specificity 95%	Advantages: establishment of a gold standard. Varied pediatric age range Limitations: human labeling subjectivity. Reliance on a custom-built device and a publicly available lung sound database covering all age ranges yet lacking pediatric-specific data. A majority (61%) of breath sounds being normal. No tabular data	No code and no data
Kim et al [42]	76 children with wheezing and healthy children (1-8 y; total 287: 103 wheezes and 184 unspecified sounds)	Prospective cohort study	2: wheezes versus other breath sounds	Recordings of Korean outpatients under realistic conditions. No lung sound database. Classified by 3 pediatric pulmonologists	Digital stethoscope (JABES GSTechnology)	3-layer LSTM^g^, 4-layer CNN, 4-layer CNN+tabular data, and 34-layer residual network with CBAM^h^+tabular data	Applying a 34-layer residual network with CBAM for audio data and MLP^i^ for tabular data exhibits high performance in wheeze sound detection and is suitable for clinical practice: accuracy 91.2%, sensibility 81%, AUC^j^ 89.1%, and *F*_1_-score 87.2%	Advantages: establishment of a gold standard. Combining tabular data improved the AI algorithm performance Limitations: human labeling subjectivity. Imbalanced data in the training data set. Cannot detect crackles and other sounds simultaneously, impacting diagnosis. 184 unspecified sounds	No code and no data^f^
Gelman et al [39]	951 patients with asthma and 167 healthy individuals (mo^k^-47 y; 1118)	Prospective cohort study	2: asthma and healthy	Recordings from patients at a polyclinic and children’s hospital in Russia. No lung sound database	Built-in or external microphones from a smartphone	NN^l^	Suggest a binary classification model for discriminating between patients with asthma and healthy individuals. Accuracy 89.4%, sensibility 89.3%, and specificity 86%	Advantage: varied pediatric age range Limitations: No use of a gold standard. NN trained on mixed adult (12%) and child (88%) lung sounds from oral cavity (4%), tracheal (50%), and chest (46%) recordings. No disclosure of whether patients were inpatients or outpatients. No tabular data	No code and no data

^a^Diagnoses are mentioned when provided by the authors.

^b^AI: artificial intelligence.

^c^SVM: support vector machine.

^d^CNN: convolutional neural network.

^e^StethoMe AI-specific architecture is not explicitly disclosed, but it could potentially involve deep learning, convolutional, or recurrent neural networks.

^f^Data declared available from the authors upon request but not open access.

^g^LSTM: long short-term memory.

^h^CBAM: convolutional block attention module.

^i^MLP: multilayer perceptron.

^j^AUC: area under the curve.

^k^Undisclosed minimum age of children.

^l^NN: neural networks.

Among the identified 7 studies, 4 (57%) [12,39,40,42] developed customized models for their classification tasks, while 3 (43%) [11,38,41] chose a preexisting ready-to-use model (ie, StethoMe AI, and an AI algorithm developed by Tuoxiao Intelligent Technology Company, Shanghai, China). Various model architectures were used for respiratory sound classification, such as support vector machines, neural networks (NNs), and convolutional NNs (Table 2). The algorithms were used for automatic classification of wheezes, crackles, and normal breath sounds. The performance measures varied across studies, with accuracies ranging from 57.7% to 97.7%, sensitivities ranging from 78.2% to 91%, specificities ranging from 82.2% to 97%, and *F*_1_-scores ranging from 66.4% to 87.2% (Table 2). None of the studies shared the algorithm code or data with the community, making reproducibility impossible.

Of the 9 records from gray literature search, the final sample consisted of 6 (67%) conference papers [44-49], 2 (22%) review articles [50,51], and 1 (11%) research article [52] (Multimedia Appendix 2 and Multimedia Appendix 3 [44-53]). Among the total of 201 articles assessed for eligibility across all literature resources, 120 (59.7%) were partially or fully composed of public data set repositories or multimedia resources of breath sounds to train and benchmark their AI-based lung sound algorithms or discuss their relevance in this field. These data sets and their primary features are detailed in Multimedia Appendix 4 [43,53-71]. The International Conference on Biomedical and Health Informatics 2017 Respiratory Sound Database and the Respiratory Acoustic Laboratory Environment represented the 2 most common data sets, used in 61 (50.8%) of 120 studies.

### Critical Appraisal and Synthesis of the Results

Table 3 summarizes the quality assessment scores of each dimension of the ChAMAI and the total score in each study. The average score of the included studies was 20.8 (SD 6.7), indicating overall medium quality within the lower range. All studies exhibited scores ranging from 13.5 to 31.5 (low to medium quality). Multimedia Appendix 5 [11,12,21,36-40] shows scores for each of the high- and low-priority items.

**Table 3:** Quality assessment scores of included studies according to the 6 ChAMAI (Checklist for the Assessment of Medical Artificial Intelligence) dimensions [21].

Study	Problem understanding (total score 10)	Data understanding (total score 6)	Data preparation (total score 8)	Modeling (total score 6)	Validation (total score 12)	Deployment (total score 8)	Total (n=50)
Mazić et al [38]	3.5	0	2	6	4.5	0.5	16.5
Grzywalski et al [11]	5.5	1	0	6	1	1	14.5
Kevat et al [12]	6.5	5	2	4	0.5	1	19
Zhang et al [41]	9	5	1	6	1	2	24
Cheng et al [40]	9	6	2	6	2	1.5	26.5
Kim et al [42]	9	5	3	6	7	1.5	31.5
Gelman et al [39]	6	0	1	5	1	0.5	13.5

### SWOT Analysis

**Figure 2:**
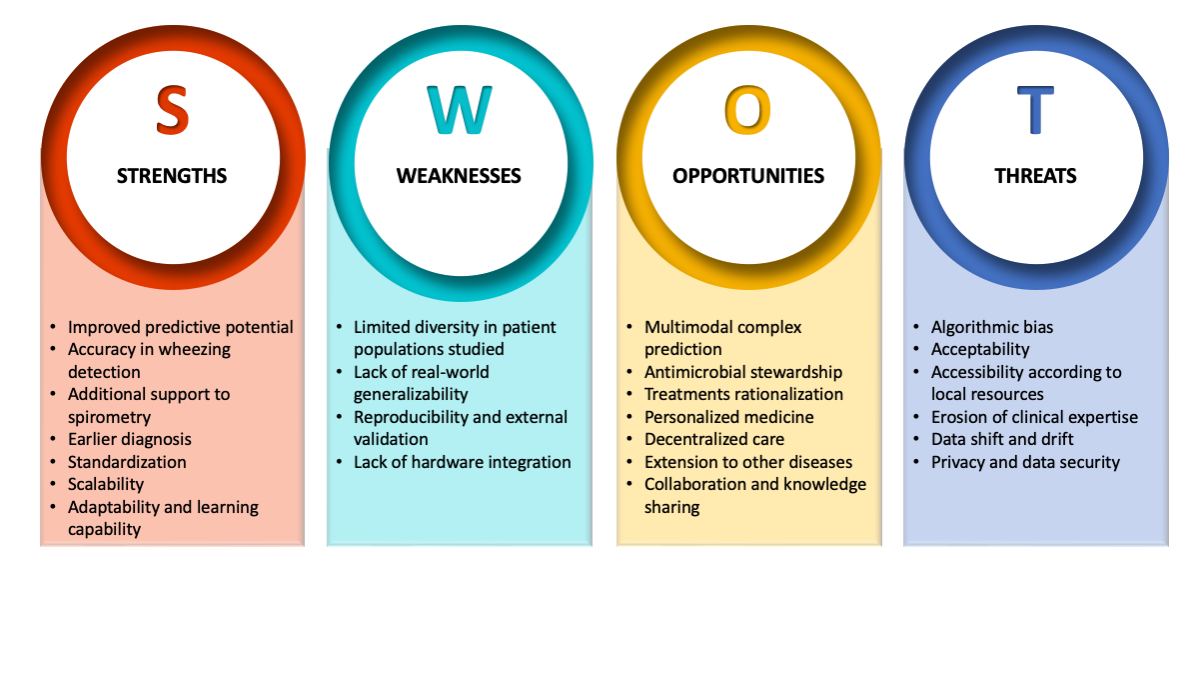
Strengths, weaknesses, opportunities, and threats (SWOT) analysis of artificial intelligence (AI)-assisted lung sound analysis for children with asthma.

A visual representation of the SWOT elements related to AI-assisted lung auscultation for pediatric asthma is presented in Figure 2. It provides a comprehensive understanding of the multifaceted impact of integrating AI into pediatric asthma care, highlighting strength and growth potential, while addressing existing challenges and threats. A detailed breakdown of the SWOT identified in the retrieved academic literature is provided in Multimedia Appendix 6 [11,12,38-42].

## Discussion

### Principal Findings

This scoping review provides an overview of the current state of research on AI models for lung sound analysis in pediatric asthma and wheezing disorders. Only 7 (11%) of the 64 studies qualified for review, highlighting the nascent stage of research in this field. Variations among studies can be attributed to heterogeneity in study populations, design, sample sizes, lung sound recording methods, and models used. Notably, although these models showed promising results in distinguishing between wheezing, crackles, and normal breath sounds, none of the studies conducted external validation to assess the reproducibility and generalizability of the model. Furthermore, the lack of open-source code makes reproducibility or external validation impossible.

In terms of quality, significant disparities were observed among the identified studies, which can be characterized as average quality overall. While problem understanding and modeling were satisfying across all studies, with the exception of the study by Mazić et al [38], there were shortcomings in adequately defining participant inclusion and exclusion criteria. Moreover, descriptions related to data preparation, validation, and deployment exhibited areas that could benefit from improvement. Therefore, further research and refinement of AI algorithms are imperative to elevate their performance and enhance their applicability across various pediatric populations and respiratory conditions. These advances will contribute to achieving more accurate and reliable AI-assisted lung sound analysis in pediatric asthma.

### Strengths

#### Improved Predictive Potential

The ability of deep learning to extract predictive patterns from complex medical data has proven utility across several domains of health care [72,73]. As shown in the above scoping review, one of the strengths of AI-based lung sound analysis is its accuracy in the detection of wheezing. These results are in line with the growing evidence found in adult studies on asthma [74].

#### Additional Support in Assessing Asthma Attack Severity Alongside Spirometry

Spirometry is considered the gold standard for assessing airway obstruction. However, performing it in young patients shows more limitations in their capacity for reproducible and analyzable voluntary breathing maneuvers than that of older individuals [75]. Conducting AI-powered auscultation before and after bronchodilator treatment in young patient population offers the potential to provide valuable additional information beyond the scope of spirometry alone. This could include the detection of subtle changes in breath sounds indicative of airway constriction or responsiveness to bronchodilators, thereby influencing treatment decisions.

#### Earlier Diagnosis

Among children with persistent, mild-to-moderate asthma, 75% have shown abnormal patterns of lung function growth and increased risk for fixed airflow obstruction and possibly chronic obstructive pulmonary disease in early adulthood [76]. Thus, AI may facilitate timely and precise identification of asthma exacerbations in children, particularly focusing on the detection of the onset of symptoms and worsening of respiratory distress within the home environment [77]. Through frequent monitoring of breath sounds, AI-powered auscultation can alert caregivers to subtle changes indicative of impending acute asthma exacerbation episodes, thereby allowing for timely proactive intervention before symptoms worsen and better management strategies [78]. This proactive approach not only improves treatment effectiveness but also encourages parents or caregivers to apply necessary preventive measures, such as adjusting medication dosages or avoiding triggers [79]. Consequently, it reinforces asthma management, helping to better control persistent asthma and minimizing further lung damage, thereby enhancing the overall quality of life of affected pediatric patients [80].

#### Standardization

Interuser variability is one of the most problematic issues in the manual assessment of lung auscultation. Standardizing lung sound analysis through AI may reduce the variability and favor more objective and targeted care in children with asthma.

#### Scalability

The rising prevalence of asthma [81] requires resources capable of adapting to respiratory epidemics [82,83]. Leveraging open-source and open-access AI algorithms allows for cost-free replication, addressing scalability concerns. Moreover, the convergence of competitively priced digital stethoscopes and AI powered by deep NNs significantly enhances diagnostic accuracy and the scalability of respiratory health care.

#### Adaptability

While humans rapidly show limited and declining performance when overwhelmed by data [84], AI models feed on large volumes of data, to which they continuously adapt by improving through iterative training and feedback loops. Algorithms can be updated to evolving data (albeit requiring a nonnegligible data pipeline for continuous learning).

### Weaknesses

#### Limited Diversity in Patient Populations Studied

While strengths focus on the potential for improved predictive performance, the limited number of patients and settings make it difficult to assess if applicable to other populations and environments because both model accuracy and performance can be compromised. In our scoping review, most of the studies showed a questionable variety, with small numbers of relatively homogeneous patient cohorts used to train the algorithms.

#### Real-World Generalizability

Studies collect data in study conditions, which may use specially trained researchers to acquire the data in conditions that do not reflect real-world environments (eg, carefully measured systematic acquisition in quiet rooms on compliant patients). Thus, algorithms might lose their predictive potential under the strain of real-world conditions. Furthermore, as in the study from Cheng et al [40], many studies assessing AI model performances often use publicly available databases (eg, the Respiratory Acoustic Laboratory Environment repository [43], the International Conference on Biomedical and Health Informatics [53], etc) to train their algorithms. However, these databases present fundamental flaws in the data collection process, such as the lack of standardization of the recording environment or the use of different stethoscopes, generating systematic biases in the robustness of their predictive and classification capacity.

#### Reproducibility and External Validation

As mentioned, no studies performed external validation (testing on a data set representing a new patient population), and none have released their data or models. Thus, making it impossible to assess the validity of the findings.

#### Lack of Hardware Integration

Accurate collection and standardization of lung audio data require acquisition by digital stethoscopes. To date, few of these devices have built-in AI to assist interpretation.

### Opportunities

#### Multimodal Complex Prediction

The etiology of asthma exacerbations is highly multifactorial, ranging from environmental and genetic factors to the immunological status of the affected child [85]. Predictive patterns are too complex to be assessed without computational assistance. The development of expert-based AI algorithms could help clinicians to identify childhood asthma more quickly, with better reproducibility and affordability [86]. New methods in multimodal decision support [87] allow algorithms to consider diverse data sources at once including imagery, sound, and clinical records. As outlined by Drummond et al [88], the implementation of digital twin systems in health care, illustrated by patient-specific virtual counterparts, integrates multiple data sources to adapt and provide better asthma management in real time. This innovative approach stands to gain from the inclusion of AI-powered lung auscultation as one of its sources in the multimodal approach. The composability of these elements could optimize medical resources to assist children in the daily management of their asthma.

#### Antimicrobial Stewardship

When faced with diagnostic uncertainty, clinicians tend to err on the side of caution. This behavior is a major issue for antibiotic stewardship and respiratory disease is the leading cause of antibiotic mismanagement. Therefore, an algorithm permitting to enhance the diagnosis of different respiratory conditions could assist in reducing the overuse of antibiotics or steroids [89].

#### Personalization

As asthma is a chronic condition, it is a good candidate for granular patient-level longitudinal data that could inform on personal evolution. Personalized medicine is a concept based on the following three elements: (1) choosing the right treatment option, (2) adapting it to the right patient, and (3) administering it at the right time [15]. Regarding asthma, the advent of personalized medicine coupled with the digitalization of data should enable specialists to develop a protocol tailored to each patient, with beneficial perspectives in empowerment and disease management [90].

#### Decentralization

Thanks to advances in cloud computing, telemedicine, and mobile health, patient data can be made available to remote care providers or algorithms. This accessibility can promote equitable access to advanced diagnostic tools and specialized expertise. The opportunity of this is especially obvious when considering resource constraints faced during the peaks of the COVID-19 pandemic. Telemedicine could allow clinicians to safely follow patients at any time regardless of the epidemiological context, thereby maintaining quality, safety, and continuity of care. Digital stethoscopes associated with telemedicine are promising areas of research and development, with many devices already designed and certified to record lung sounds [91]. Digitization and automation played a major role during the COVID-19 pandemic and now offer additional benefits for flexibility and more equitable access to predictive medicine [92,93]. Finally, in low- and middle-income countries, where asthma management and control are frequently insufficient [94], automated interpretation of clinical examinations holds the potential to fill resource gaps, especially in remote settings which have sparse medical expertise or not enough trained staff.

#### Extension to Other Diseases

While wheezing seems to be a strong signal, automated interpretation of lung sounds has been shown to be promising in various respiratory diseases [9,95]. For instance, a recent multicenter study [13] has shown the performances of a single deep-learning algorithm to detect the audio signatures of several pediatric respiratory conditions, including pneumonia, wheezing respiratory disorders, and bronchiolitis from lung auscultation performed in real clinical conditions and low-resources countries.

#### Collaboration

By enabling collaboration and knowledge sharing between institutions and the medical community, AI models promote a collaborative environment that favors a collective understanding of respiratory conditions, stimulates joint research efforts, and accelerates the advancement of innovative solutions to improve patient care. New technologies enabling collaborative learning without needing to share data are particularly promising. Like federated learning, where, instead of sharing the data to the source of the model, the model is shared to the source of the data [96].

### Threats

#### Bias

AI-based tools have been evaluated for their inherent risk of bias, such as underdiagnosis of underrepresented populations [97] or algorithmic bias, which challenge the implementation of these new tools. Undetected biases may result from poor-quality or biased selection of the original data. It is essential to ensure representative data collection, large volumes of data to train the algorithms, robust external validation, and close interdisciplinary collaboration to avoid bias. These measures, combined with good user training, the development of transparent and interpretable AI models, the conduct of comprehensive clinical validation studies, and the establishment of clear guidelines, should also address users’ concerns about AI-assisted pulmonary auscultation.

#### Acceptability

The use of AI in medicine is still a new concept to much of the general public and patients (as well as clinicians) may not feel comfortable using or interpreting AI-powered devices. In the case of pediatric asthma, a French study found that the parental perception of AI in parents of patients with asthma was mostly positive. Parents acknowledge the potential of AI to assist physicians in diagnosing and determining the most appropriate treatment, with >50% of them agreeing to adhere to an algorithmic decision system for managing their child’s asthma [98]. Furthermore, since the COVID-19 pandemic, parents have shown an increased preference for AI-based management. Interestingly, children seem even more willing to accept AI-based asthma management than their parents [99]. By contrast, the acceptability of AI is contingent on the need for AI systems to be finely tuned and updated regularly. This requirement ensures that AI remains accurate, reliable, and aligned with evolving data and user expectations, which contributes to its continued adoption and trustworthiness.

#### Accessibility

Seamless integration into existing health care systems, interoperability, and ease of use of AI models require careful planning, adequate resources, and collaboration between technology developers and local health care providers. These considerations are particularly relevant when it comes to equitably addressing the needs of disadvantaged populations or those living in low-income countries where, ironically, asthma prevalence is high [81]. Digital health may offer opportunities to achieve universal health [100]. Nevertheless, these prospects are hindered by a shortage of medical technologies, insufficient digital infrastructure for their support and maintenance, limited financial resources, and low educational levels [101], all of which collectively exacerbate the digital divide.

#### Clinical Expertise

While the integration of AI into digital lung auscultation analysis offers valuable insights and can enhance diagnostic performances, there is a need to question the adequacy of health care professionals’ training in the era of AI-driven auscultation. Relying solely on automated algorithms for this essential task could unwittingly erode the proficiency of clinicians in recognizing subtle nuances and contextual factors useful in making accurate diagnoses. Striking a balance between AI support and maintaining the auscultatory skills of medical practitioners should be a priority in the evolving landscape of digital health.

#### Data Shift and Drift

As clinical AI systems become more common, it raises questions about the readiness of clinicians to identify issues when these systems fail. One common issue is data shift, where AI underperforms when there is a mismatch between the data used to train the AI model and the data it encounters in the real world [102]. For example, during the COVID-19 pandemic, a widely used sepsis-alerting AI system had to be deactivated because it could not adapt to changing patient demographics. Data drift, on the other hand, refers to the gradual change in data patterns over time, leading to outdated and less accurate AI models. These threats can result in biased predictions and impair the classification model’s performance. To mitigate these threats, AI models need to be constantly monitored and adapted to changes in the associated data to ensure their long-term reliability and effectiveness. Nonetheless, clinicians will need to remain vigilant and report discrepancies between their clinical judgment and AI predictions.

#### Privacy and Data Security

Finally, 1 of the main threats concerns ethical considerations relating to privacy and data security, given that the use of AI algorithms relies on accessing and processing sensitive patient information. Encryption of data transit is crucial for patient confidentiality and user trust, with access to authorized individuals only [103]. Strikingly, none of the threats and challenges mentioned earlier were addressed, or only to a very limited extent, in the studies identified in our scoping review, as shown by the poor rating of the Deployment dimension of the ChAMAI.

### Limitations

This study has limitations. First, we did not conduct a systematic review. Given the novelty of AI in the field of lung auscultation and the rapid evolution of techniques, a scoping review was preferred to a traditional systematic one for a preliminary assessment of a potentially large and diverse body of literature on this broad topic and its application to the pediatric population [104]. Similarly, a formal meta-analysis was not feasible due to the paucity and heterogeneity of available data. Instead, by synthesizing existing evidence in a charting format, this scoping review provides a comprehensive overview of the research landscape, identifies key themes, and highlights areas to inform the decision-making process for further investigation and potentially guide the design of future systematic reviews and meta-analyses. A second limitation relates to the potential omission of significant research related to the use of AI in children with asthma. This has been minimized by an exhaustive exploration of the main medical literature databases, a meticulous analysis of the references within the selected articles, and a double assessment of the identified studies. However, our inclusion criteria may have led to the unintentional exclusion of information. Third, as AI-powered lung sound analysis is an emerging topic and studies take time to be published, studies or gray literature may have been published after the study inclusion date, potentially leading to incomplete results. Finally, using the ChAMAI tool to assess the quality of the selected studies could be criticized. Nevertheless, to the best of our knowledge, this tool is the most comprehensive and advanced tool to date for such an assessment.

### Conclusions

This scoping review and the accompanying SWOT analysis provide a comprehensive assessment of the existing literature on the use of AI-assisted lung auscultation in children aged <18 years with asthma or wheezing disorders. The findings highlight the potential benefits of integrating AI algorithms in objective lung sound evaluation, offering improved diagnostic accuracy, personalized management strategies, and remote monitoring capacities. Addressing the identified limitations, promoting standardization, and addressing ethical considerations will be critical for the successful implementation of AI-based systems in pediatric asthma care, ultimately improving the outcome of affected children. Further research and collaborations are necessary to deploy the full potential of AI in revolutionizing the diagnosis and management of pediatric asthma.

## Appendix

Multimedia Appendix 1 Population-Concept-Context (PCC) framework for identifying the main concepts of the scoping review.

Multimedia Appendix 2 Distribution of article types according to Google Scholar occurrences (N=183).

Multimedia Appendix 3 Characteristics of individual sources of evidence from gray literature search.

Multimedia Appendix 4 Public repositories of breath sounds: resources for artificial intelligence–based lung sound algorithm training and benchmarking.

Multimedia Appendix 5 Quality assessment of selected studies using the Checklist for the Assessment of Medical Artificial Intelligence.

Multimedia Appendix 6 Strengths, weaknesses, opportunities, and threats of retrieved academic literature.

Multimedia Appendix 7 PRISMA-ScR checklist.

## Abbreviations

AI: artificial intelligence
ChAMAI: Checklist for the Assessment of Medical Artificial Intelligence
MR: major revision
mR: minor revision
NN: neural network
PRISMA-ScR: Preferred Reporting Items for Systematic Reviews and Meta-Analyses extension for Scoping Reviews
SWOT: strengths, weaknesses, opportunities, and threats

## Data Availability

All data analyzed during this study are included in this published paper and its supplementary information files.
