# Supplementary material for: Automated Interpretation of Lung Sounds by Deep Learning in Children With Asthma: Scoping Review and Strengths, Weaknesses, Opportunities, and Threats Analysis
Source: J Med Internet Res. 2024 Aug 23;26:e53662. doi: 10.2196/53662 (PMC11380063; doi:10.2196/53662)
Supplement: Multimedia Appendix 1 [file jmir_v26i1e53662_app1.pdf]

Multimedia Appendix 1. Population-Concept-Context (PCC) framework for identifying the main concepts of the scoping review.

**PCC Element**	Definition
**Population**	• Children with asthma and wheezing disorders.
**Concept**	• Strengths, weaknesses, opportunities, and threats of automated lung sound analysis using artificial intelligence to detect or classify asthma.
**Context**	• Research articles within the last 23 years (2000-2023), published in English.
