# Supplementary material for: Automated Interpretation of Lung Sounds by Deep Learning in Children With Asthma: Scoping Review and Strengths, Weaknesses, Opportunities, and Threats Analysis
Source: J Med Internet Res. 2024 Aug 23;26:e53662. doi: 10.2196/53662 (PMC11380063; doi:10.2196/53662)
Supplement: Multimedia Appendix 2 [file jmir_v26i1e53662_app2.pdf]

Multimedia Appendix 2. Distribution of article types according to Google Scholar occurrences (N=183)

**Article type**	Number identified, n(%)	Duplicate already identified in the academic literature search	Number included, n(%)
**Research article**	90 (49.1)	4	1 (0.5)
**Conference paper**	54 (29.5)	-	6 (3.3)
**Review**	34 (18.6)	-	2 (1.1)
**Thesis**	7 (3.8)	-	0
**Book chapter**	6 (3.3)	-	0
**Online document**	1 (0.5)	-	0
