# Supplementary material for: Automated Interpretation of Lung Sounds by Deep Learning in Children With Asthma: Scoping Review and Strengths, Weaknesses, Opportunities, and Threats Analysis
Source: J Med Internet Res. 2024 Aug 23;26:e53662. doi: 10.2196/53662 (PMC11380063; doi:10.2196/53662)
Supplement: Multimedia Appendix 3 [file jmir_v26i1e53662_app3.pdf]

Multimedia Appendix 3. Characteristics of individual sources of evidence from grey literature search

Study	Article type	Summary
[1]	Research article	Developed using a custom-built digital stethoscope, an AI-based binary classifier (Feedforward NN) achieved an accuracy of 96.8%, sensitivity of 93.8%, and positive predictive value of 96.8% in distinguishing 32 children with asthma from 63 healthy individuals (aged 0-11 years) based on hospital recordings from Taiwan.
[2]	Conference paper	This paper explores the efficiency of 7 AI classification algorithms (SVM, k-NN, NN, RF, LR, and NB) on web-based publicly available phonopneumograms versus hospital recordings from Croatian children aged one to six, emphasizing SVM and k-NN as the most successful classifiers with 99% accuracy, especially on hospital signals under realistic conditions.
[3]	Conference paper	This paper explores the stratified analysis of adventitious respiratory sounds event classification in the ICBHI 2017 respiratory sound database [4]. Sounds from subjects of varying ages, diseases, body mass indexes, and recorded with diverse equipment are analyzed, with a focus on understanding model behavior for each subpopulation. Three machine learning algorithms (SVM, RUSBoost, CNN) classified the test set, addressing two binary classification problems (crackles vs. others, wheezes vs. others) and one 3-class problem (crackles vs. wheezes vs. others). Healthy subjects were excluded from analysis due to limited annotated data. Overall, the CNNs achieved the best results, except in cases with sparse data where SVM or RUSBoost models achieved better results. The CNN model, while highly effective, requires ample data for optimal performance, while SVM and RUSBoost models can achieve strong results with less data, albeit with a feature extraction stage.
[5]	Conference paper	This paper comprehensively reviews technologies for developing asthmatic wheeze detection systems, focusing on their functionalities and effectiveness. Neural networks exhibit superior accuracy, with specific emphasis on Kuo et al.'s pediatric study [1], the only reference concerning children in this paper.
[6]	Conference paper	This paper explores a system for recognizing and classifying children's respiration patterns. It includes collecting breath sound signals and introducing a time-varying acoustic feature. The proposed classifiers categorize the breath sounds based on the features of interest. Clinical data validated the approaches, sourced from two Chinese and one US hospitals, encompassing children aged 2 months to 6 years with asthma, croup, pneumonia, or in a healthy state. Additionally, an ANN was utilized for breath sound classification.
[7]	Conference paper	This paper explores CNN's use in detecting adventitious sounds, utilizing two datasets: the ICBHI 2017 public database with 126 subjects and a recorded pediatric auscultation dataset with 222 subjects of undisclosed age. The CNN's detection performance is assessed using these datasets individually and in combination. Results indicate higher accuracy for the ICBHI database compared to the pediatric database and the mixed databases. The study underscores the potential of CNN in adventitious respiratory sound detection but suggests further improvements are needed, particularly for pediatric and mixed datasets.
[8]	Conference paper	This paper explores different methods to analyze lung sounds and extract distinctive features then classify them to diagnose lung sounds in infants and children, aiming to categorize sounds as normal, wheeze, or stridor. Features were extracted using DWT, STFT, and MFCCs, coupled with classifiers including ANN, SVM, k-NN, and NB. Using 300 lung sounds database from a children hospital in Egypt, with an additional 146 wheezes for validation from young infants (undisclosed age till the age of 12 years old), the study identifies DWT combined with ANN as the most accurate method across all categories, with MFCC performing well in wheeze detection. DWT and MFCC outperform STFT.
[9]	Systematic review	This paper provides a systematic review of computer-based respiratory sound analysis techniques, initially identifying over 120 articles. After screening, 55 articles were selected, of which 2 focus on image processing methods. The review covers various time frames without specific date ranges. Research on lung sound analysis is categorized into visual analysis, statistical methods, and machine learning, with machine learning comprising 33 articles (none were included in our scoping review). Overall, the review highlights the potential of computer-based approaches to enhance diagnostic accuracy for respiratory diseases in both clinical and research settings.
[10]	Review	This systematic review and meta-analysis examined studies utilizing computerized lung sound analysis to detect abnormal lung sounds in specific respiratory disorders. A search across multiple databases yielded 208 articles, with 8 selected for review (none were included in our scoping review). Most studies utilized electret microphones or piezoelectric sensors for auscultation, alongside Fourier Transform and NN algorithms for analysis. The meta-analysis indicated an overall sensitivity of 80% (95%, CI 72–86%) and specificity of 85% (95%, CI 78–91%) for computerized lung sound analysis in detecting wheezes or crackles. Despite limited data quality, computerized lung sound analysis shows promise, particularly in providing high specificity for identifying abnormal lung sounds.

ANN, Artificial Neural Network; CI, Confidence Interval; CNN, Convolutional Neural Network; DWT, Discrete Wavelet Transform; ICBHI, International Conference on Biomedical and Health Informatics; k-NN, k-nearest neighbor; LR, Logistic Regression; MFCC, Mel-Frequency Cepstral Coefficients; NB, Naive Bayes; N/A, Non-Available; NN, Neural Network; PPV, Positive Predictive Value; RF, Random Forests; RUSBoost, Random Undersampling Boosting; STFT, Short Time Fourier Transform; SVM, Support Vector Machine

1. Kuo HC, Lin BS, Wang YD, Lin BS. Development of automatic wheeze detection algorithm for children with asthma. IEEE Access. 2021;9:126882-90. doi: 10.1109/ACCESS.2021.3111507.
2. Milicevic M, Mazic I, Bonkovic M, editors. Classification accuracy comparison of asthmatic wheezing sounds recorded under ideal and real-world conditions. 2016.
3. Fernandes T, Rocha BM, Pessoa D, de Carvalho P, Paiva RP, editors. Classification of adventitious respiratory sound events: a stratified analysis. 2022 IEEE-EMBS International Conference on Biomedical and Health Informatics (BHI); 2022: IEEE.
4. Rocha B, Filos D, Mendes L, Vogiatzis I, Perantoni E, Kaimakamis E, et al., editors. A respiratory sound database for the development of automated classification. Precision Medicine Powered by pHealth and Connected Health: ICBHI 2017, Thessaloniki, Greece, 18-21 November 2017; 2018: Springer.
5. Uwanthika I, Madushanka P, Deraniyagala D. Identification of related technologies associated with asthmatic wheeze detection systems: A Review2023.
6. Liu L, Li W, Jiang C, editors. Time-varying respiratory feature recognition and classification for respiration health and disease screening in children. 2021 IEEE International Conference on Systems, Man, and Cybernetics (SMC); 2021: IEEE.
7. Liu R, Cai S, Zhang K, Hu N, editors. Detection of adventitious respiratory sounds based on convolutional neural network. 2019 International Conference on Intelligent Informatics and Biomedical Sciences (ICIIBMS); 2019: IEEE.
8. Gouda A, El Shehaby S, Diaa N, Abougabal M, editors. Classification techniques for diagnosing respiratory sounds in infants and children. 2019 IEEE 9th Annual Computing and Communication Workshop and Conference (CCWC); 2019: IEEE.
9. Palaniappan R, Sundaraj K, Ahamed NU, Arjunan A, Sundaraj S. Computer-based respiratory sound analysis: a systematic review. IETE Technical Review. 2013;30(3):248-56.
10. Gurung A, Scrafford CG, Tielsch JM, Levine OS, Checkley W. Computerized lung sound analysis as diagnostic aid for the detection of abnormal lung sounds: a systematic review and meta-analysis. Respir Med. 2011 Sep;105(9):1396-403. PMID: 21676606. doi: 10.1016/j.rmed.2011.05.007.
