# Supplementary material for: Automated Interpretation of Lung Sounds by Deep Learning in Children With Asthma: Scoping Review and Strengths, Weaknesses, Opportunities, and Threats Analysis
Source: J Med Internet Res. 2024 Aug 23;26:e53662. doi: 10.2196/53662 (PMC11380063; doi:10.2196/53662)
Supplement: Multimedia Appendix 4 [file jmir_v26i1e53662_app4.pdf]

Database or audio sources	Country of origin	n (%)	No. of subjects ([a]), [age range in years], F:M[b]	No. of audio samples	Access mode	Audio acquisition equipment	Abnormal lung sounds labeled	Lung diseases labeled (No. of subjects)
ICBHI (International Conference on Biomedical and Health Informatics) 2017 Respiratory Sound Database [1]	Portugal, Greece	50 (27.3)	126 (26), [0.25-93], 79:46	920	Online repository	3M Littmann Classic II SE<br>3M Littmann 3200<br>Welch Allyn Meditron Elite<br>AKG C417L microphone	Wheezes (886), Crackles (1864), Wheezes + Crackles (506)	Asthma (1), COPD (64), Bronchiolitis (6), Bronchiectasis (7), URTI (14), LRTI (2), Pneumonia (6)
R.A.L.E. (Respiratory Acoustic Laboratory Environment)[2]	Canada	10 (5.5)	70 (17)	>50	Online repository	Siemens EMT25C contact accelerometers<br>Sony ECM140 microphone	Wheezes (252), Crackles (70), Ronchi, Squeaks, Squawks, Pleural rubs	Asthma, COPD, Bronchiolitis, Bronchogenic carcinoma, Lung fibrosis, Cystic fibrosis
KAUH database [3]	Jordan	5 (2.7)	112 (35), [12-90], 43:69	308	Online repository	3M Littmann 3200	Wheezes (41), Crackles (8), Wheezes + Crackles (2), Crepitations (23), Bronchial (1), Bronchial + Crackles (2)	Asthma (32), Bronchitis (3), COPD (9), Heart failure (21), Lung fibrosis (5), Pleural effusion (2), Pneumonia (5)
3M Littmann Stethoscope Lung Sound Library [4]	USA	5 (2.7)	-	-	Mobile app	-	-	-
ETSU (East Tennessee State University) repository [5]	USA	5 (2.7)	-	20	Online repository	-	Wheezes, Crackles, Pleural rub, Stridor, Rhonchi	-
Stethographics lung sound samples	USA	5 (2.7)	-	-	Non publicly available	-	-	-
Lippincott NursingCenter [6]	USA	4 (2.2)	-	-	Online repository	-	Wheezes, Coarse and fine crackles, Stridor, Rhonchi	-
Thinklabs Lung Sound Library [7]	USA	4 (2.2)	-	-	Online repository	Thinklabs stethoscopes	Wheezes, Crackles, Rhonchi, Stridor, Pleural rubs	Asthma, Bronchiolitis, COPD, Laryngomalacia, Pulmonary edema
Medscape [8]	USA	4 (2.2)	-	-	Online repository	-	-	-
Wikipedia [9]	USA	4 (2.2)	-	-	Online repository	-	-	-
EasyAuscultation [10]	USA	4 (2.2)	-	-	Online repository	-	Wheezes, Coarse and fine crackles, Rhonchi, Stridor, Pleural rubs	-
Reference Database of Respiratory Sounds [11]	Europe	4 (2.2)	-	-	Online repository	-	-	-
CSU (Colorado State University)[c]	USA	4 (2.2)	-	-	Online repository	-	-	-
SPRSound [12]	China	2 (2.2)	292 (20), [0.2-16.2], 140:152	2683	Online repository	Digital stethoscope (Yunting model II)	Wheezes (865), Rhonchi (53), Stridor (17), Coarse crackles (66), Fine crackles (1167), Wheezes + Crackles (34)	Asthma (33), Bronchitis (19), Pneumonia (197), Other diseases (23)

HF_Lung_V1 database [13]	Taiwan	2 (2.2)	279 (0), >20yr, gender ratio N/A	9765	Online repository	Stethoscope (3M Littmann 3200); A customized multichannel acoustic recording device (HF-Type-1)	Wheezes (8457), Crackles (15606), Ronchi (4740), Stridor (686)	Acute and chronic respiratory failure, COPD, Emphysema, Pneumonia,
RDA (Respiratory and Drug Actuation) Benchmark and Dataset [14]	Greece	1 (0.5)	3 adults	370	Online repository	Microphone attached to an inhalation device	193 drug actuation segments, 319 inhalation, 620 exhalation and 505 environmental noise segments	-
MARS (Marburg Respiratory Sound) [15]	Germany	1 (0.5)	390	5000	Online repository	Experimental recording device	Wheezes, Fine and coarse crackles, Bronchial, Stridor, Ronchi	Asthma (50) COPD (40), Lung fibrosis (5), Pneumonia (45)
M3DICINE Lung Sound Dataset [16]	Australia	1 (0.5)	78, children and adults	224		M3DICINE's Stethee Pro device	-	-
ASTRA database [17]	France	1 (0.5)	-	-	CD-ROM	-	-	-
Understanding Lung Sounds [18]	USA	1 (0.5)	-	-	CD-ROM	-	-	-
Understanding Heart Sounds and Murmurs: with an Introduction to Lung Sounds [19]	USA	1 (0.5)	-	-	CD-ROM	-	-	-
Fundamentals of Lung and Heart Sounds [20]	USA	1 (0.5)	-	-	CD-ROM	-	-	-

ICBHI, International Conference on Biomedical and Health Informatics; KAUH, King Abdullah University Hospital; N/A, Not Available; LRTI, Lower Respiratory Tract Infections; R.A.L.E., Respiration Acoustic Laboratory Environment; URTI, Upper Respiratory Tract Infections
[a] Number of healthy individuals
[b] Female (F) to Male (M) ratio
c Database's web link no longer accessible

References:

1. Rocha B, Filos D, Mendes L, Vogiatzis I, Perantoni E, Kaimakamis E, et al., editors. A respiratory sound database for the development of automated classification. Precision Medicine Powered by pHealth and Connected Health: ICBHI 2017, Thessaloniki, Greece, 18-21 November 2017; 2018: Springer.
2. Owens D. Rale lung sounds 3.0. CIN: Computers, Informatics, Nursing. 2002;5(3):9-10.
3. Fraiwan M, Fraiwan L, Khassawneh B, Ibnian A. A dataset of lung sounds recorded from the chest wall using an electronic stethoscope. Data Brief. 2021 Apr;35:106913. PMID: 33732827. doi: 10.1016/j.dib.2021.106913.
4. 3M Littmann. Available from: https://www.littmann.in/3M/en_IN/littmann-stethoscopes-in/education/training/.
5. East Tennessee State University, pulmonary breath sounds. Available from: http://faculty.etsu.edu/arnall/www/public_html/heartlung/breathsounds/contents.html.

6. Lippincott NursingCenter. Available from: https://www.nursingcenter.com/ncblog/october-2016/breath-sounds-test-your-knowledge.
7. Thinklabs One Lung Sounds Library. Available from: https://www.thinklabs.com/sound-library.
8. Medscape. Available from: https://emedicine.medscape.com/article/1894146-overview?form=fpf#a3
9. Wikipedia. Available from: https://en.wikipedia.org/wiki/Respiratory_sounds.
10. EasyAuscultation. Available from: https://www.easyauscultation.com/heart-lung-sounds-reference-guide/pulmonic.
11. Reference Database of Respiratory Sounds. Available from: https://www.ers-education.org/e-learning/reference-database-of-respiratory-sounds/other/.
12. Zhang Q, Zhang J, Yuan J, Huang H, Zhang Y, Zhang B, et al. SPRSound: open-source SJTU paediatric respiratory sound database. IEEE Trans Biomed Circuits Syst. 2022 Oct;16(5):867-81. PMID: 36070274. doi: 10.1109/TBCAS.2022.3204910.
13. Hsu FS, Huang SR, Huang CW, Huang CJ, Cheng YR, Chen CC, et al. Benchmarking of eight recurrent neural network variants for breath phase and adventitious sound detection on a self-developed open-access lung sound database-HF_Lung_V1. PLoS One. 2021;16(7):e0254134. PMID: 34197556. doi: 10.1371/journal.pone.0254134.
14. Respiratory and Drug Actuation Dataset. IEEE Dataport. 2022.
15. Gross V, Hadjileontiadis LJ, Penzel T, Koehler U, Vogelmeier C, editors. Multimedia database "Marburg Respiratory Sounds (MARS)". Proceedings of the 25th Annual International Conference of the IEEE Engineering in Medicine and Biology Society (IEEE Cat No03CH37439); 2003 17-21 Sept. 2003.
16. Fernando T, Sridharan S, Denman S, Ghaemmaghami H, Fookes C. Robust and interpretable temporal convolution network for event detection in lung sound recordings. IEEE J Biomed Health Inform. 2022 Jul;26(7):2898-908. PMID: 35061595. doi: 10.1109/JBHI.2022.3144314.
17. Racineux J-L. Auscultation pulmonaire. [Rueil-Malmaison]: AstraZeneca respiratoire [Rueil-Malmaison]; 2007.
18. Lehrer S. Understanding lung sounds: Steven Lehrer; 2018. ISBN: 1981284060.
19. Tilkian AG, Conover MB. Understanding heart sounds and murmurs: with an introduction to lung sounds. 4th edition ed: Saunders; 2001 January 15, 2011. ISBN: 978-0721676432.
20. Wilkins RL, Hodgkin JE, Lopez B. Fundamentals of lung and heart sounds: Mosby; 2004. ISBN: 999763683X.
