# Supplementary material for: Automated Interpretation of Lung Sounds by Deep Learning in Children With Asthma: Scoping Review and Strengths, Weaknesses, Opportunities, and Threats Analysis
Source: J Med Internet Res. 2024 Aug 23;26:e53662. doi: 10.2196/53662 (PMC11380063; doi:10.2196/53662)
Supplement: Multimedia Appendix 5 [file jmir_v26i1e53662_app5.pdf]

	Authors						
**Requirements, according to [21]**	**Mazic [38]**	**Grzywalski [11]**	**Kevat [12]**	**Zhang [41]**	**Cheng [40]**	**Kim [42]**	**Gelman [39]**
Problem understanding							
**1. Is the study population described, also in terms of inclusion/exclusion criteria?**	1	0	1	2	2	1	0
**2. Is the study design described?**	0	0	0	1	1	2	2
3. Is the study setting described?	0,5	1	0,5	1	1	1	1
**4. Is the source of data described?**	2	2	2	2	2	2	2
**5. Is the medical task reported?**	0	2	2	2	2	2	0
6. Is the data collection process described, also in terms of setting-specific data collection strategies?	0	0,5	1	1	1	1	1
Data understanding							
**7. Are the subject demographics described in terms of average age; age variability; gender; comorbidities; ethnicity; socioeconomic status?**	0	0	2	2	2	1	0
**8. If the task is supervised, is the gold standard described?** ***Number of annotators producing the labels; their profession and expertise; instructions given for quality control; inter-rater agreement score; labelling technique***	0	1	1	2	2	2	0
**9. In the case of tabular data, are the features described?**	0	0	2	1	2	2	0

Data preparation							
**10. Is outlier detection and analysis performed and reported?**	0	0	0	0	0	0	0
**11. If applicable, is missing-value management described?**	0	0	0	0	0	0	0
**12. Is feature pre-processing performed and described?**	2	0	2	1	2	2	0
**13. Is data imbalance analysis and adjustment performed and reported?**	0	0	0	0	0	1	1
Modeling							
**14. Is the model task reported?**	2	2	2	2	2	2	2
**15. Is the model output specified?**	2	2	2	2	2	2	1
**16. Is the model architecture or type described?**	2	2	0	2	2	2	2
Validation							
**17. Is the data splitting described?**	2	0	0	0	0	2	1
**18. Is the model training and selection described? (range, selection and specification of hyper-parameters)**	1	0	0	0	1	2	0
**19. (classification models) Is the model calibration described?**	0	0	0	0	0	0	0
**20. Is the internal validation procedure described?**	1	0	0	0	0	2	0
21. Has the model been externally validated?	0	0	0,5	0	0	0	0
**22. Are the main error-based metrics used?**	0	1	0	1	1	1	0

23. Are some relevant errors described?	0,5	0	0	0	0	0	0
Deployment							
24. Is the target user indicated?	0	1	1	1	1	0,5	0,5
25. (classification models) Is the utility of the model discussed?	0	0	0	1	0	0	0
26. Is information regarding model interpretability and explainability available?	0,5	0	0	0	0	0,5	0
27. Is there any discussion regarding model fairness, ethical concerns or risks of bias?	0	0	0	0	0,5	0,5	0
28. Is any point made about the environmental sustainability of the model, or about the carbon footprint, of either the training phase or inference phase (use) of the model?	0	0	0	0	0	0	0
**29. Is code and data shared with the community?**	0	0	0	0	0	0	0
30. Is the system already adopted in daily practice?	0	0	0	0	0	0	0
**Total:**	**16,5**	**14,5**	**19**	**24**	**26,5**	**31,5**	**13,5**

No concern

Minor concern

Major concern

Items in **bold** indicate priority aspects to be considered
