# Supplementary material for: Automated Interpretation of Lung Sounds by Deep Learning in Children With Asthma: Scoping Review and Strengths, Weaknesses, Opportunities, and Threats Analysis
Source: J Med Internet Res. 2024 Aug 23;26:e53662. doi: 10.2196/53662 (PMC11380063; doi:10.2196/53662)
Supplement: Multimedia Appendix 6 [file jmir_v26i1e53662_app6.pdf]

Multimedia appendix 6. Strengths, weaknesses, opportunities and threats (SWOT) of retrieved academic literature.

Article	Strengths	Weaknesses	Opportunities	Threats
Mazić [1]	• Adaptable to 0-6 years old unlike spirometers and peak flow meters<br>• Discrimination of wheeze versus non wheeze even in sounds containing artifacts	• Suboptimal quality of the training set can lead to misclassifications	• More respiratory sounds acquisition<br>• Cascade structure algorithm to be tested	---
Grzywalski [2]	• Very efficient tool for pathological sound detection: after initial requirements, the neuronal network algorithm can match or even outperform human performance	---	• Valuable support for physicians, medical students, or care providers<br>• Medical training and education<br>• Hospital and home monitoring<br>• Patient safety (reaction speed)<br>• Reduction of treatment costs	---
Kevat [3]	• Equal or better performance of AI algorithms than expert humans	• Device-dependent differences in accuracy	• Improvement of diagnostic accuracy<br>• Tracking and prediction of patient improvement or deterioration over time<br>• Assessment of AI use in real-world clinical environments	---
Zhang [4]	• Higher accuracy in the recognition of adventitious breath sounds than that of general pediatricians, junior residents, and most specialists except pulmonologists<br>• Saving, sharing and remote transmission	• Lack of an objective gold standard for the evaluation of breath sounds<br>• Accuracy of the algorithm limited to the age range of the target group in the derivation algorithm	• Telemedicine sessions<br>• Chronic disease follow-up for families<br>• Improvement of lung auscultation skills<br>• Remote analysis<br>• Improvement of diagnostic ability<br>• Improvement of treatment decision making	---
Cheng [5]	• Strong agreement between trained expertise and AI	• No readily available clinically verified pediatric breath sound database	• Cloud-based algorithm technology with smartphones connected to digital stethoscopes<br>• Telemedicine<br>• Signal sound amplification<br>• Ambient noise reduction<br>• Creation of a public repository of pediatric normal and adventitious breath sounds	---
Kim [6]	• High agreement with classification by conventional auscultation<br>• Performance of the AI model, which outperforms clinician's auscultation	• Few studies verifying models that classify breathing sounds from actual clinical situations<br>• Few studies with pediatric patients	---	---

Gelman [7]	• High performance of the model in clinical practice, with high sensitivity when the level of pathological sound is low and cannot be discerned by the human ear<br>• Reduction of subjective assessment<br>• Performance improved with tabular data addition (age, gender)	• Small sample size, with need for data augmentation<br>• Binary classification model<br>• Large amount of real-world data needs to be collected through multicenter prospective studies	• Development of deep-learning model applied to various breathing sounds<br>• Computer-aided control over the patient's condition and efficiency of medication in real time<br>• Diagnosis and monitoring in children < 5 years in whom physical examination and spirometry can be challenging, in remote areas and/or in patients outside the hospital or for telemedicine applications	---

References

1. Mazić I, Bonković M, Džaja B. Two-level coarse-to-fine classification algorithm for asthma wheezing recognition in children's respiratory sounds. Biomedical Signal Processing and Control. 2015;21:105-18.
2. Grzywalski T, Piecuch M, Szajek M, Breborowicz A, Hafke-Dys H, Kocinski J, et al. Practical implementation of artificial intelligence algorithms in pulmonary auscultation examination. Eur J Pediatr. 2019 Jun;178(6):883-90. PMID: 30927097. doi: 10.1007/s00431-019-03363-2.
3. Kevat A, Kalirajah A, Roseby R. Artificial intelligence accuracy in detecting pathological breath sounds in children using digital stethoscopes. Respir Res. 2020 Sep 29;21(1):253. PMID: 32993620. doi: 10.1186/s12931-020-01523-9.
4. Zhang J, Wang HS, Zhou HY, Dong B, Zhang L, Zhang F, et al. Real-world verification of artificial intelligence algorithm-assisted auscultation of breath sounds in children. Front Pediatr. 2021;9:627337. PMID: 33834010. doi: 10.3389/fped.2021.627337.
5. Cheng ZR, Zhang H, Thomas B, Tan YH, Teoh OH, Pugalenthi A. Assessing the accuracy of artificial intelligence enabled acoustic analytic technology on breath sounds in children. J Med Eng Technol. 2022 Jan;46(1):78-84. PMID: 34730469. doi: 10.1080/03091902.2021.1992520.
6. Kim BJ, Kim BS, Mun JH, Lim C, Kim K. An accurate deep learning model for wheezing in children using real world data. Sci Rep. 2022 Dec 28;12(1):22465. PMID: 36577766. doi: 10.1038/s41598-022-25953-1.
7. Gelman A, Furman EG, Kalinina NM, Malinin SV, Furman GB, Sheludko VS, et al. Computer-aided detection of respiratory sounds in bronchial asthma patients based on machine learning method. Sovrem Tekhnologii Med. 2022;14(5):45-51. PMID: 37181833. doi: 10.17691/stm2022.14.5.05.
